# L-PGDS–PGD2–DP1 Axis Regulates Phagocytosis by CD36^+^ MGs/MΦs That Are Exclusively Present Within Ischemic Areas After Stroke

**DOI:** 10.3390/cells13201737

**Published:** 2024-10-20

**Authors:** Takayuki Nakagomi, Aya Narita, Hideaki Nishie, Akiko Nakano-Doi, Toshinori Sawano, Yu Fukuda, Tomohiro Matsuyama

**Affiliations:** 1 Institute for Advanced Medical Sciences, Hyogo Medical University, 1-1 Mukogawa-cho, Nishinomiya 663-8501, Japan; nari1119@hyo-med.ac.jp (A.N.); nakano@hyo-med.ac.jp (A.N.-D.); 2 Department of Therapeutic Progress in Brain Diseases, Hyogo Medical University, 1-1 Mukogawa-cho, Nishinomiya 663-8501, Japan; tomohiro@hyo-med.ac.jp; 3 Nippon Zoki Pharmaceutical Co., Ltd., 4-2-3 Hirano-machi, Chuo-ku, Osaka 541-0046, Japan; h-nishie@nippon-zoki.co.jp (H.N.); y-fukuda@nippon-zoki.co.jp (Y.F.); 4 Department of Biomedical Sciences, Ritsumeikan University, 1-1-1 Nojihigashi, Kusatsu 525-8577, Japan; t-sawano@fc.ritsumei.ac.jp

**Keywords:** ischemic stroke, pericyte, lipocalin-type prostaglandin D synthase, prostaglandin D2, microglia/macrophages, CD36, scavenger, phagocytosis

## Abstract

Brain injuries, such as ischemic stroke, cause cell death. Although phagocytosis of cellular debris is mainly performed by microglia/macrophages (MGs/MΦs), excessive accumulation beyond their phagocytic capacities results in waste product buildup, delaying brain cell regeneration. Therefore, it is essential to increase the potential for waste product removal from damaged brains. Lipocalin-type prostaglandin D synthase (L-PGDS) is the primary synthase for prostaglandin D2 (PGD2) and has been reported as a scavenger of waste products. However, the mechanism by which the L-PGDS–PGD2 axis exerts such an effect remains unelucidated. In this study, using a mouse model of ischemic stroke, we found that L-PGDS and its downstream signaling pathway components, including PGD2 and PGD2 receptor DP1 (but not DP2), were significantly upregulated in ischemic areas. Immunohistochemistry revealed the predominant expression of L-PGDS in the leptomeninges of ischemic areas and high expression levels of DP1 in CD36^+^ MGs/MΦs that were specifically present within ischemic areas. Furthermore, PGD2 treatment promoted the conversion of MGs/MΦs into CD36^+^ scavenger types and increased phagocytic activities of CD36^+^ MGs/MΦs. Because CD36^+^ MGs/MΦs specifically appeared within ischemic areas after stroke, our findings suggest that the L-PGDS–PGD2–DP1 axis plays an important role in brain tissue repair by regulating phagocytic activities of CD36^+^ MGs/MΦs.

## 1. Introduction

Ischemic stroke is a critical disease that causes brain cell death. Following stroke, microglia/macrophages (MGs/MΦs) accumulate within the necrotic tissues, serving as scavengers by phagocytizing cellular debris [1]. Although MGs/MΦs are considered the principal cells involved in waste product clearance, it remains unclear how MG/MΦ-derived phagocytic activities are regulated in injured brains.

Lipocalin-type prostaglandin D synthase (L-PGDS, also known as prostaglandin D2 synthase) is a protein mainly distributed in the brain. L-PGDS functions as a chaperone for waste products in the brain, such as amyloid-β [2,3] and biliverdin [4], suggesting that it plays an important role in various brain diseases, such as Alzheimer’s disease (AD) and brain hemorrhage. Although the exact roles of L-PGDS in ischemic stroke pathogenesis remain unclear, mice lacking L-PGDS were reported to show negative outcomes, with enlarged infarcted areas compared to control mice [5]. The precise origin and phenotypes of L-PGDS-producing cells in the brain have yet to be elucidated. However, L-PGDS is strongly expressed on the surface of the brain, such as the leptomeninges [6]. Furthermore, we previously demonstrated that the leptomeninges, which cover the brain’s surface, were closely associated with vascular pericytes throughout the brain. Because brain pericytes were resistant to ischemia and survived within ischemic areas even after permanent ischemic stroke [7,8,9], they may serve as L-PGDS-producing cells in ischemic areas following stroke.

L-PGDS has multiple functions, including converting prostaglandin H2 (PGH2) into prostaglandin D2 (PGD2) [10]. The PGD2 levels are significantly increased in brains with pathological conditions, such as AD [11], traumatic brain injury [12], and ischemic stroke [13,14,15]. PGD2 binds to PGD2 receptor 1 (DP1, also known as prostaglandin D2 receptor [PTGDR] or PTGDR1) and PGD2 receptor 2 (DP2, also known as PTGDR2) [16]. DP1 is expressed in various brain cells, including MGs/MΦs [17,18,19], astrocytes [20], and neurons [21]. DP2 is expressed in astrocytes [17] but not in MGs/MΦs [22]. Considering that neurons and astrocytes are vulnerable to ischemia/hypoxia and that MGs/MΦs are predominant in ischemic areas after stroke [7], it is possible that DP1, rather than DP2, plays a key role in ischemic areas. Supporting our hypothesis, DP1 knockout mice were reported as susceptible to ischemic brain injuries [23], and stimulation with DP1 receptor-selective agonist BW245C improved neurological functions and reduced the ischemic volumes in mice following stroke [24,25]. However, the mechanism by which the L-PGDS–PGD2–DP1 axis exerts positive effects on brains with pathological conditions remains unclear.

In the present study, using a mouse model of ischemic stroke, we found that the L-PGDS–PGD2–DP1 axis was activated and DP1 was expressed in CD36^+^ MGs/MΦs within ischemic areas. Additionally, PGD2 promoted the conversion of MGs/MΦs into CD36^+^ scavenger types and increased the phagocytic activities of CD36^+^ MGs/MΦs. Collectively, these results support the novel finding that the L-PGDS–PGD2–DP1 axis modulates brain repair processes by regulating phagocytic activities of CD36^+^ MGs/MΦs.

## 2. Materials and Methods

### 2.1. Animal Studies and Preparation of Brain Tissue Samples

Permanent focal cerebral ischemia was induced in 6–10-week-old male adult CB-17/Icr-+/+Jcl mice (Clea Japan Inc., Tokyo, Japan) by middle cerebral artery occlusion (MCAO). Briefly, the mice underwent isoflurane anesthetization, and MCAO was performed by electrocoagulation, followed by disconnection of the left MCA, as previously described [7,26,27,28]. This strain of mice showed highly reproducible infarction size, as described previously [7,26].

To collect brain tissue samples, the mice were anesthetized with a mixture of medetomidine, midazolam, and butorphanol before performing transcardial perfusion with 4% paraformaldehyde (PFA), as previously described [26,27,28]. After fixation in 4% PFA for 24 h, the brain tissue samples were placed in 30% sucrose for cryoprotection and frozen at −80 °C. Then, brain tissue sections (20-μm coronal sections) were stained to assess the lipid content using an Oil Red O Stain Kit (Abcam, Cambridge, UK), according to the manufacturer’s instructions.

In another set of experiments, after fixation with 4% PFA for 24 h, brain tissue samples were processed for paraffin embedding. Then, brain tissue sections (8-μm coronal sections) underwent immunohistochemical staining.

All animal experiments were approved by the Animal Care Committee of Hyogo Medical University (approval numbers: 22-052A and 23-007AG).

### 2.2. Immunohistochemistry

The brain tissue sections were deparaffinized and underwent heat treatment using a microwave for epitope retrieval in citrate buffer solution (pH 6.0; Abcam) for 10 min. Then, the samples were incubated with primary antibodies against L-PGDS (1:100, mouse; Santa Cruz Biotechnology, Dallas, TX, USA), glial fibrillary acidic protein (GFAP; 1:1000, rabbit; Abcam), platelet-derived growth factor receptor-β (PDGFRβ; 1:200, goat; R&D Systems, Minneapolis, MN, USA), DP1 (1:200, rabbit; Arigo Biolaboratories Corp., Hsinchu, Taiwan), ionized calcium-binding adapter molecule 1 (Iba1; 1:500, goat; LSBio, Shirley, MA, USA, and 1:500, rabbit; Wako, Osaka, Japan), CD45 (1:200, rat; Thermo Fisher Scientific, Waltham, MA, USA), CD36 (1:100, goat; R&D Systems), and myelin basic protein (MBP; 1:100, mouse; R&D Systems). After washing in PBS, the sections were stained using Alexa Fluor 488- or 555-conjugated secondary antibodies (1:500; Molecular Probes, Eugene, OR, USA). Cell nuclei were counterstained with 4′,6-diamidino-2-phenylindole (DAPI; 1:500; Kirkegaard & Perry Laboratories, Inc., Gaithersburg, MD, USA). Images were recorded using an LSM780 confocal laser microscope (Carl Zeiss AG, Oberkochen, Germany).

In another set of experiments, brain sections (8-μm coronal sections) were incubated with a primary antibody against CD36 (1:100, goat; R&D Systems) after deparaffinization and heat treatment using a microwave for epitope retrieval. Then, they were stained with 3,3′-diaminobenzidine tetrahydrochloride (Vector Laboratories Inc., Burlingame, CA, USA), and the cell nuclei were counterstained with hematoxylin. Images were captured under light microscopy (Olympus, Tokyo, Japan) using a digital camera system, as previously described [7].

### 2.3. Cell Culture

To investigate whether ischemic injury promoted L-PGDS production, commercially available mouse brain vascular pericytes (#M1200, ScienCell Research Laboratories, Carlsbad, CA, USA) were incubated with supernatant obtained from ischemic areas. Briefly, brain tissues were isolated from ischemic areas on day 7 after stroke. After adjusting to a concentration of 10 mg/mL in Dulbecco’s Modified Eagle’s Medium/Nutrient Mixture F-12 (DMEM/F12; Thermo Fisher Scientific), the tissue samples were homogenized and centrifuged at 1000 rpm for 5 min. Then, the supernatant was collected and frozen at −80 °C before use. Brain pericytes (3 × 10^4^ cells/cm^2^) were inoculated into DMEM/F12 supplemented with 2% fetal bovine serum (FBS) in poly-L-lysine-coated dishes. After 24 h, the medium was replaced with fresh DMEM/F12, and 300 µL of the previously prepared supernatant was added. The brain pericytes were incubated for 3 days and collected for quantitative reverse transcription-polymerase chain reaction (RT-qPCR) analysis to determine the L-PGDS expression levels.

Commercially available mouse brain MGs/MΦs (#SCC134, EMD Millipore Corporation, Temecula, CA, USA) (5 × 10^3^ cells/cm^2^) were inoculated into dishes containing DMEM (Thermo Fisher Scientific) supplemented with 10% FBS and 2 mM L-glutamine (EMD Millipore). After 24 h, the medium was replaced with fresh DMEM supplemented with 2% FBS. Then, MGs/MΦs were treated with 2.5 µM PGD2 (MedChemExpress, Monmouth Junction, NJ, USA) or dimethyl sulfoxide (DMSO) as the vehicle control, followed by further incubation for 3 days. The cells were collected and subjected to microarray analysis.

In another set of experiments, MGs/MΦs were treated with DMSO (control) or different doses of PGD2 (0.1, 0.5, or 2.5 µM) and incubated for 3 days. Then, the collected cells underwent RT-qPCR and Western blot (WB) analysis.

To evaluate the phagocytic activities of MGs/MΦs, they were treated with DMSO (control) or PGD2 (2.5 µM) for 3 days, followed by incubation with sicastar-greenF microbeads (diameter: 300 nm; micromod Partikeltechnologie GmbH, Rostock, Germany) for 2 h. Then, the MGs/MΦs were collected, fixed, immunostained with CD11b antibody (1:100; BD Biosciences, Franklin Lakes, NJ, USA), and subjected to fluorescence-activated cell sorting (FACS) analysis.

In some experiments, 100 µM sulfo-N-succinimidyl oleate (SSO) (Cayman Chemical, Ann Arbor, MI, USA) was added to the medium containing PGD2-treated MGs/MΦs 1 h before microbead treatment to inhibit CD36 function.

### 2.4. WB Analysis

WB analysis was performed as previously described [7]. Briefly, brain tissue samples were obtained from the MCA area of sham-operated mice or ischemic areas of mice at 1, 3, 5, or 7 days after MCAO. In another set of experiments, brain tissue samples were obtained from ipsilateral ischemic areas or contralateral nonischemic MCA areas at 7 days after MCAO (3 samples/area, 3 animals/area [*n* = 3]). Then, the total protein in the brain tissue samples was extracted using RIPA buffer (Thermo Fisher Scientific), and the protein concentration was measured using a Pierce BCA Protein Assay Kit (Thermo Fisher Scientific). In another experiment, 1 × 10^6^ cultured cells were treated with RIPA buffer.

The prepared brain tissue samples (3 µg/lane) or cultured cells (10 µg/lane) underwent sodium dodecyl sulfate-polyacrylamide gel electrophoresis, and the separated proteins were transferred onto polyvinylidene difluoride membranes (Bio-Rad, Hercules, CA, USA). The membranes were incubated with primary antibodies against L-PGDS (1:1000, mouse; Santa Cruz Biotechnology), CD36 (1:2000, goat; R&D Systems), and β-actin (1:2000, mouse; Sigma-Aldrich, St. Louis, MO, USA), followed by incubation with horseradish peroxidase-labeled secondary antibodies (1:2000, mouse; Cell Signaling Technology, Danvers, MA, USA, and 1:1000, goat; Santa Cruz Biotechnology). The membranes were stripped before treating with β-actin, and the expression levels of the target protein (L-PGDS, CD36) and β-actin were evaluated using the same membrane. The protein bands labeled with the antibodies were detected using an enhanced chemiluminescence reagent (Chemi-Lumi One, Nacalai Tesque, Kyoto, Japan), according to the manufacturer’s instructions. The expression levels of L-PGDS and CD36 were normalized to β-actin using ImageJ analysis software, as previously described [7].

### 2.5. Reverse Transcription Quantitative-Polymerase Chain Reaction

Total RNA was isolated from brain tissue samples obtained from ipsilateral ischemic areas or contralateral nonischemic MCA areas at 7 days after MCAO (3 samples/area, 3 animals/area [*n* = 3]) using a RNeasy Mini Kit (Qiagen, Hilden, Ger many). Then, complementary DNA was synthesized using SuperScript IV Reverse Transcriptase (Thermo Fisher Scientific), and qPCR was performed using TB Green Fast qPCR Mix (Takara Bio Inc., Shiga, Japan), according to the manufacturer’s instructions. The primer sequences were as follows: L-PGDS: 5′-AGTGGTGGAGGCCAACTATG-3′ (forward) and 5′-TCTCCTTCAGCTCGTCCTTC-3′ (reverse); DP1: 5′-ATGAACGAGTCCTATCGCTGT-3′ (forward) and 5′-ACACGAGCACATAAAAGACCG-3′ (reverse); DP2: 5′-AGATGGTCCAGCTTCCAAACC-3′ (forward) and 5′-ACAGGATGAGTCCGTTTTCCA-3′ (reverse); CD36: 5′-GATTAATGGCACAGACGCAGC-3′ (forward) and 5′-TTCAGATCCGAACACAGCGT-3′ (reverse); β-actin: 5′-CGCGAGCACAGCTTCTTTG-3′ (forward) and 5′-CGTCATCCATGGCGAACTGG-3′ (reverse). The relative level of mRNA expression was determined using the ∆∆CT method, and the expression level was normalized to that of β-actin.

### 2.6. Flow Cytometry Analysis

MGs/MΦs were labeled with APC-conjugated antibody against CD36 (1:100; BD Biosciences) and underwent flow cytometry analysis using a LSRFortessa X-20 FACS device (BD Biosciences), as previously described [29]. The ratio of fluorescein isothiocyanate (FITC)^+^ MGs/MΦs between DMSO-treated (control) and PGD2-treated (2.5 µM) cells (3 samples/treatment, *n* = 3) was determined to evaluate the movement of microbeads labeled with green signal into MGs/MΦs. Additionally, after PGD2 (2.5 µM) treatment, the ratio of CD36^+^FITC^+^ MGs/MΦs was evaluated between the DMSO-treated cells (control) and SSO-treated cells (3 samples/treatment, *n* = 3).

### 2.7. Enzyme-Linked Immunosorbent Assay

Enzyme-linked immunosorbent assay (ELISA) was performed to evaluate the amount of PGD2 in the brain tissue samples. Briefly, brain tissue samples were obtained from ipsilateral ischemic areas or contralateral nonischemic MCA areas at 7 days after MCAO (3 samples/area, 3 animals/area [*n* = 3]). Then, the PGD2 concentration within ischemic areas or contralateral MCA areas was measured using a Mouse PGD2 ELISA Kit (CUSABIO, Houston, TX, USA), which employs a competitive inhibition enzyme immunoassay technique, according to the manufacturer’s protocol. Each sample was measured twice, and the mean value was calculated using a standard curve. The PGD2 concentration in ischemic areas was normalized to that of the contralateral MCA areas.

### 2.8. Electron Microscopy

Mice at 7 days post-stroke were placed under deep anesthesia and perfused with a phosphate buffer solution containing 2% PFA and 2% glutaraldehyde. Then, brain tissues (*n* = 3) were collected from ischemic areas for electron microscopy analysis, as previously described [29].

### 2.9. Microarray Analysis

Total RNA was isolated from MGs/MΦs treated with the vehicle (control) or PGD2 (2.5 µM) for 3 days using a RNeasy Mini Kit. Then, the samples (*n* = 1 per group) underwent microarray analysis, and the resultant data were analyzed using Affymetrix Transcriptome Analysis Console software, as previously described [29].

### 2.10. Statistical Analysis

Data were presented as means ± standard deviations. Differences between two groups were evaluated using Student’s *t*-test. Comparisons among three or more groups were performed using a one-way analysis of variance, followed by post hoc tests. *p*-values < 0.05 were considered statistically significant.

## 3. Results

### 3.1. L-PGDS Expression Increases in Ischemic Areas After Stroke

**Figure 1:**
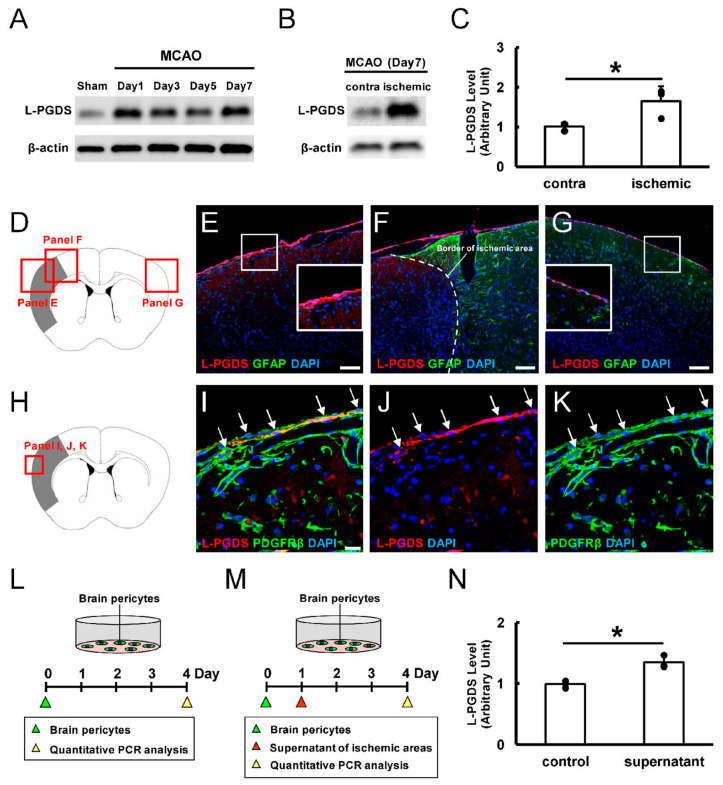
(**A**) L-PGDS expression by WB from samples collected from MCA areas of sham-operated mice or ischemic areas at 1, 3, 5, and 7 days post-stroke. (**B**,**C**) WB analysis at 7 days post-stroke. L-PGDS expression levels were significantly higher in ischemic areas compared to the contralateral side of nonischemic areas. (**D**–**G**) Immunohistochemistry for L-PGDS and GFAP. L-PGDS was strongly detected in the leptomeninges of ischemic areas (L-PGDS [(**E**–**G**): red]); GFAP [(**E**–**G**): green]; DAPI [(**E**–**G**): blue]). (**H**–**K**) Immunohistochemistry for L-PGDS and PDGFRβ. L-PGDS was co-expressed in PDGFRβ^+^ pericytes in the leptomeninges of ischemic areas ((**I**–**K**), arrows) (L-PGDS [(**I**,**J**): red]; PDGFRβ [(**I**,**K**): green]; DAPI [(**I**–**K**): blue]). (**L**–**N**) RT-qPCR analysis. L-PGDS levels were significantly higher in brain pericytes treated with supernatant from ischemic areas (**M**,**N**) than in untreated pericytes (control) (**L**,**N**). Scale bars: (**E**–**G**) 100 µm; (**I**) 20 µm. * *p* < 0.05 between the contralateral side of nonischemic areas and ischemic areas, *n* = 3 for each area (**C**). * *p* < 0.05 between the control and supernatant–treated group, *n* = 3 for each group (**N**). Abbreviations: DAPI, 4′,6-diamidino-2-phenylindole; GFAP, glial fibrillary acidic protein; L-PGDS, lipocalin-type prostaglandin D synthase; MCA, middle cerebral artery; PDGFRβ, platelet-derived growth factor receptor-β; RT-qPCR, quantitative reverse transcription-polymerase chain reaction; WB, Western blot.

We first investigated gene expression levels of L-PGDS after ischemic stroke by subjecting mice to MCAO. Then, brain tissue samples were collected from ischemic areas on days 1, 3, 5, and 7 post-stroke. As a control, brain tissue samples were collected from nonischemic areas of sham-operated mice. WB analysis showed that, compared to the control, L-PGDS expression increased in mice after ischemic stroke (Figure 1A). We further investigated differences in L-PGDS expression between ischemic areas and the contralateral side of nonischemic areas at 7 days post-stroke. WB analysis revealed that L-PGDS expression was significantly higher in ischemic areas compared to the contralateral side of nonischemic areas (Figure 1B,C). Thus, L-PGDS was upregulated in ischemic areas after stroke.

To further investigate the localization of L-PGDS^+^ cells in ischemic areas, we performed double staining immunohistochemistry for L-PGDS and GFAP. Consistent with previous studies [6], L-PGDS was present in the leptomeninges (Figure 1D–G). Notably, L-PGDS was strongly observed in the leptomeninges of ischemic areas lacking GFAP expression (Figure 1E) compared to the contralateral nonischemic areas (Figure 1G). Because the leptomeninges are closely associated with blood vessels, partially as pericytes throughout the brain [9,30], we further investigated whether L-PGDS was expressed in brain pericytes of post-stroke leptomeninges (Figure 1H–K). Immunohistochemistry revealed that L-PGDS^+^ cells in the leptomeninges of ischemic areas co-expressed the pericytic marker PDGFRβ (Figure 1I–K). In contrast, L-PGDS was rarely observed in PDGFRβ^+^ pericytes of brain parenchyma in the ischemic areas (Figure 1I–K). Thus, L-PGDS was mainly expressed in the leptomeninges of ischemic areas, including pericytic cells.

To investigate the mechanism of L-PGDS upregulation in pericytes after ischemic stroke, brain pericytes were cultured in medium without or with the supernatant extracted from the tissues of ischemic areas (Figure 1L,M, respectively). The supernatant addition increased L-PGDS expression in brain pericytes compared to brain pericytes cultured in medium without supernatant addition (control) (Figure 1N). Thus, ischemia activated L-PGDS expression in brain pericytes.

### 3.2. Higher Levels of PGD2 Are Present in Ischemic Areas After Stroke

**Figure 2:**
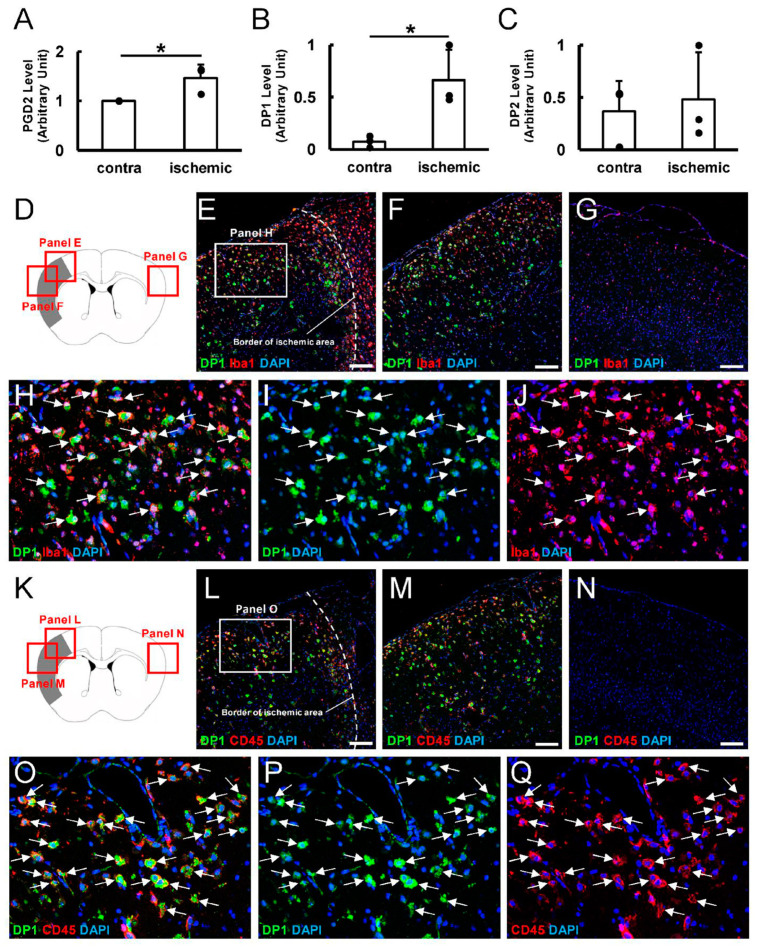
(**A**) ELISA analysis at 7 days post-stroke. PGD2 levels were significantly higher in ischemic areas compared to the contralateral side of nonischemic areas. (**B**,**C**) RT-qPCR analysis at 7 days post-stroke. DP1 levels were significantly higher in ischemic areas than in the contralateral side of nonischemic areas (**B**), while differences in DP2 levels were not significant between the two areas (**C**). (**D**–**J**) Immunohistochemistry for DP1 and Iba1. DP1 was largely expressed in Iba1^+^ cells within ischemic areas ((**E**,**F**,**H**–**J**) [arrows]) but not in Iba1^+^ cells in the contralateral side of nonischemic areas (**G**) (DP1 [(**E**–**I**): green]; Iba1 [(**E**–**H**,**J**): red]; DAPI [(**E**–**J**): blue]). (**K**–**Q**) Immunohistochemistry for DP1 and CD45. Although many DP1^+^ cells within ischemic areas co-expressed CD45 ((**L**,**M**,**O**–**Q**) [arrows]), DP1^+^ or CD45^+^ cells were rarely observed in the contralateral side of nonischemic areas (**N**) (DP1 [(**L**–**P**): green]; CD45 [(**L**–**O**,**Q**): red]; DAPI [(**L**–**Q**): blue]). Scale bars: (**E**–**G**,**L**–**N**) 100 µm. * *p* < 0.05 between the contralateral side of nonischemic areas and ischemic areas (**A**,**B**). *n* = 3 for each area (**A**–**C**). Abbreviations: DAPI, 4′,6-diamidino-2-phenylindole; DP1, prostaglandin D2 receptor 1; DP2, prostaglandin D2 receptor 2; ELISA, enzyme-linked immunosorbent assay; PGD2, prostaglandin D2; RT-qPCR, quantitative reverse transcription-polymerase chain reaction.

L-PGDS is an enzyme that converts PGH2 into PGD2 [31]. Therefore, we investigated PGD2 levels in post-stroke mice using ELISA. The PGD2 levels were significantly higher in ischemic areas compared to the contralateral side of nonischemic areas of the cortex (Figure 2A). These findings were consistent with reports of significantly increased PGD2 levels in brains with pathological conditions, such as after ischemic stroke [13,14,15].

### 3.3. DP1 Expression Increases in Ischemic Areas After Stroke

Because PGD2 binds to DP1 and DP2 [16], we next investigated the gene expression levels of DP1 and DP2 using samples of brain tissue at 7 days post-stroke. RT-qPCR analysis revealed that gene expression of DP1 was significantly increased in ischemic areas compared to the contralateral side of nonischemic areas of the cortex (Figure 2B), while gene expression levels of DP2 did not differ significantly between ischemic areas and the contralateral side of the cortex (Figure 2C). These findings indicated activation of the L-PGDS–PGD2–DP1 axis in ischemic areas after stroke.

We used immunohistochemistry to further investigate DP1 localization in post-stroke brains. Immunohistochemistry (Figure 2D–J) showed that many DP1^+^ cells were observed in the ischemic areas (Figure 2E,F), but DP1^+^ cells were rarely observed in the contralateral side of nonischemic areas of the cortex (Figure 2G). Additionally, DP1^+^ cells in ischemic areas largely expressed Iba1 (Figure 2H–J). Because activated MGs/MΦs express CD45 [32], we used immunohistochemistry to investigate whether DP1^+^ cells in ischemic areas expressed CD45. Immunohistochemistry (Figure 2K–Q) showed that many CD45^+^ cells co-expressing DP1 were observed in ischemic areas (Figure 2L,M,O–Q), but CD45^+^ cells were rarely observed in the contralateral side of nonischemic areas of the cortex (Figure 2N). These observations are consistent with reports of strong DP1 expression in activated MGs/MΦs in brains with pathological conditions [18,19].

### 3.4. PGD2 Treatment Promotes the Production of CD36^+^ Scavenger Types of MGs/MΦs

Our data indicated that DP1 was predominantly expressed in MGs/MΦs within ischemic areas. Therefore, we investigated whether PGD2 treatment affected the fate of MGs/MΦs by treating brain-derived MGs/MΦs with PGD2 for 3 days. MGs/MΦs treated with DMSO were used as controls. The PGD2-treated MGs/MΦs and control MGs/MΦs were collected for microarray analysis.

**Figure 3:**
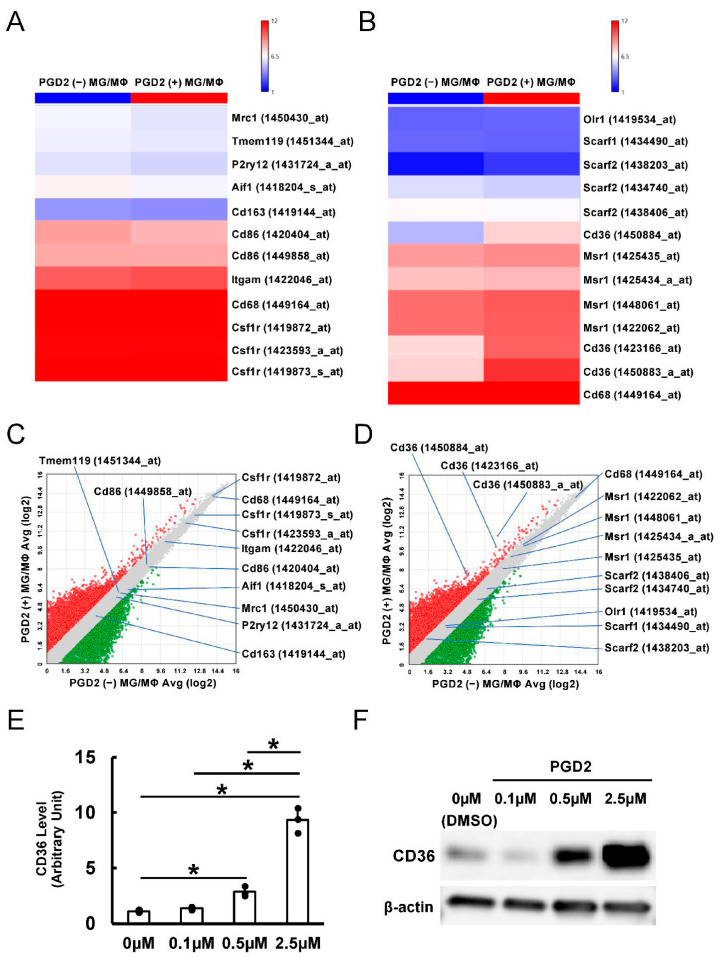
(**A**) Heatmapping analysis of the expression patterns of conventional, M1, and M2 types of MGs/MΦs treated without or with PGD2. (**B**) Heatmapping analysis of the expression patterns of scavenger receptors in MGs/MΦs treated without or with PGD2. (**C**) Scatter plot analysis of the expression patterns of conventional, M1, and M2 types of MGs/MΦs treated without or with PGD2. (**D**) Scatter plot analysis of the expression patterns of scavenger receptors in MGs/MΦs treated without or with PGD2. (**E**,**F**) RT-qPCR (**E**) and WB analysis (**F**) for CD36 levels in MGs/MΦs treated with various concentrations of PGD2. * *p* < 0.05 among groups, *n* = 3 for each group (**E**). Abbreviations: MGs/MΦs, microglia/macrophages; PGD2, prostaglandin D2; RT-qPCR, quantitative reverse transcription-polymerase chain reaction; WB, Western blot.

We first investigated the influence of PGD2 treatment on M1 and M2 marker expression in MGs/MΦs. Heatmapping (Figure 3A) and scatter plot analysis (Figure 3C) revealed that PGD2 treatment did not significantly alter the markers for conventional (Itgam, Aif1, Csf1r, P2ry12, and Tmem119); M1 (Cd68 and Cd86); and M2 (Mrc1 and Cd163) types.

We next investigated the effect of PGD2 on the expression of MGs/MΦs scavenger receptors. Heatmapping (Figure 3B) and scatter plot analysis (Figure 3D) indicated that PGD2 treatment did not significantly change the levels of Msr1 (Scara1), Cd68 (Scard1), Olr1 (Scare1), Scarf1, or Scarf2. However, the expression of microglial scavenger receptor Cd36 (Scarb3), which was reported as related to phagocytic activities in post-stroke mouse brains [33], was significantly upregulated in MGs/MΦs following PGD2 treatment. Furthermore, RT-qPCR analysis revealed that PGD2 treatment significantly increased gene expression of CD36 in a dose-dependent manner (Figure 3E). Similarly, WB analysis revealed that PGD2 treatment increased the CD36 protein levels in a dose-dependent manner (Figure 3F). These results confirmed that PGD2 promoted CD36 induction at both the gene and protein levels.

### 3.5. DP1 Is Expressed in CD36^+^ MGs/MΦs That Exclusively Appear in Ischemic Areas After Stroke

**Figure 4:**
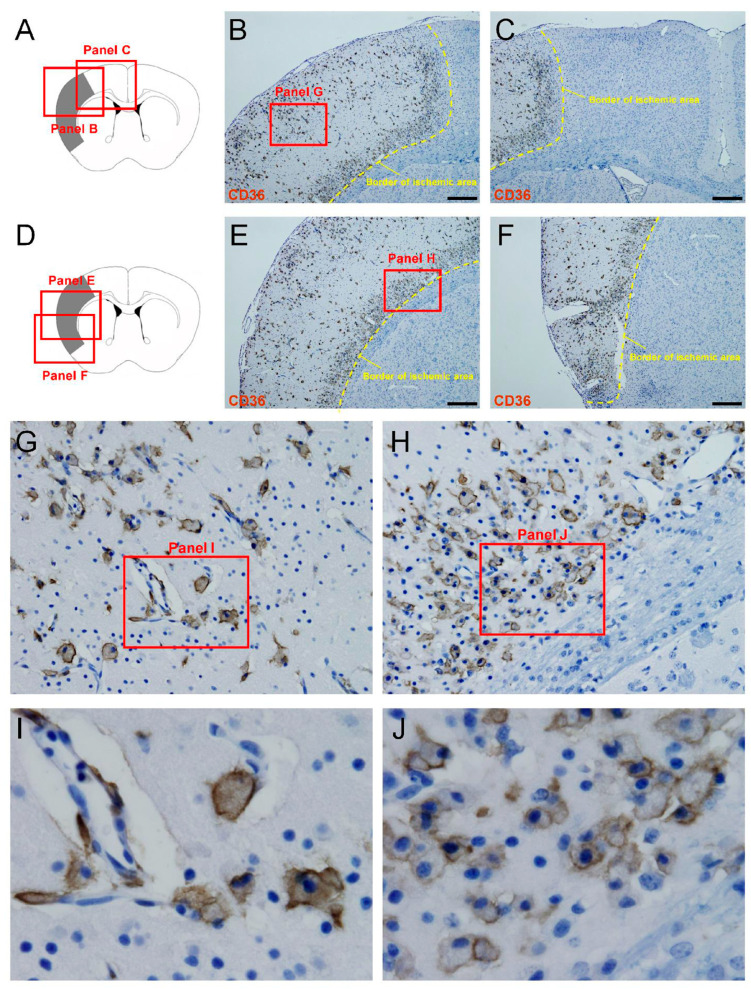
(**A**–**J**) Immunohistochemistry for CD36 at 7 days post-stroke showed that CD36^+^ cells were exclusively expressed within ischemic areas. Scale bars: (**B**,**C**,**E**,**F**) 500 µm.

We used immunohistochemistry to investigate the expression patterns of CD36^+^ cells following ischemic stroke. CD36^+^ cells were exclusively expressed within ischemic areas (Figure 4A–J).

**Figure 5:**
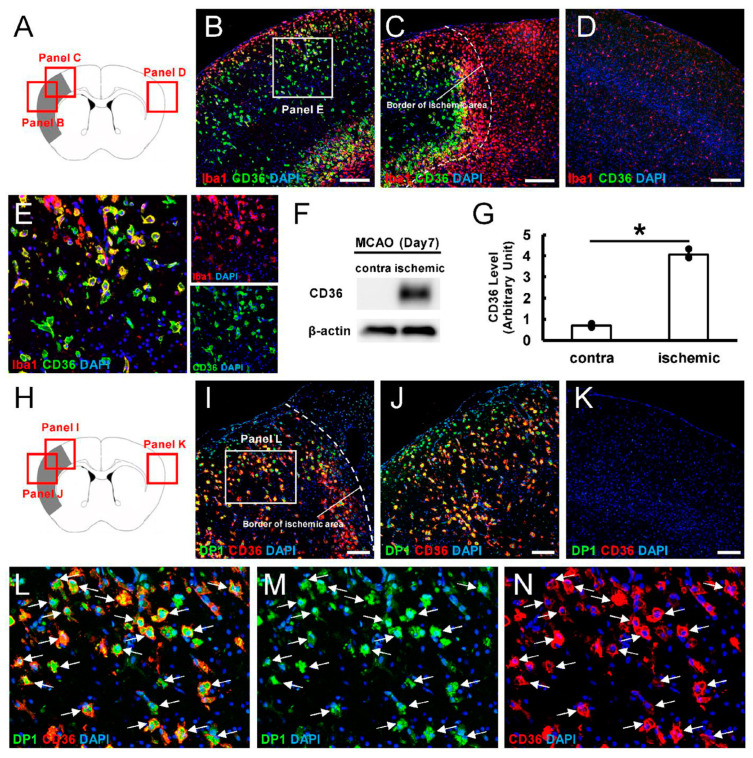
(**A**–**E**) Immunohistochemistry for Iba1 and CD36. CD36 was exclusively expressed in Iba1^+^ cells within ischemic areas (**B**,**C**,**E**), while CD36 was not observed in Iba1^+^ cells in the contralateral side of nonischemic areas (D) (Iba1 [(**B**–**E**): red]; CD36 [(**B**–**E**): green]; DAPI [(**B**–**E**): blue]). (**F**,**G**) WB analysis at 7 days post-stroke. CD36 levels were significantly higher in ischemic areas compared to the contralateral side of nonischemic areas. (**H**–**N**) Immunohistochemistry for DP1 and CD36. Although many DP1^+^ cells within ischemic areas co-expressed CD36 ((**I**,**J**,**L**–**N**) [arrows]), DP1^+^ or CD36^+^ cells were rarely observed in the contralateral side of nonischemic areas (**K**) (DP1 [(**I**–**M**): green]; CD36 [(**I**–**L**,**N**): red]; DAPI [(**I**–**N**): blue]). Scale bars: (**B**–**D**) 200 µm; (**I**–**K**) 100 µm. * *p* < 0.05 between the contralateral side of nonischemic areas and ischemic areas, *n* = 3 for each area (**G**). Abbreviations: DAPI, 4′,6-diamidino-2-phenylindole; DP1, prostaglandin D2 receptor 1; WB, Western blot.

Thus, we next performed immunohistochemistry for CD36 and Iba1 (Figure 5A–E). Although Iba1^+^ cells were observed throughout the brain, including ischemic areas (Figure 5B,C) and the contralateral side of nonischemic areas (Figure 5D), many Iba1^+^ cells were present within and around ischemic areas (Figure 5B,C). In contrast, CD36^+^ cells were absent from the contralateral side of nonischemic areas (Figure 5D), and many CD36^+^ cells, which, in part, co-expressed Iba1, were specifically observed within ischemic areas (Figure 5B,E). Consistent with these results, WB analysis revealed that, although CD36 was rarely observed in cells from the contralateral side of MCA areas, higher levels of CD36 expression were observed in cells from ischemic areas (Figure 5F,G). These findings indicated that CD36 was specifically expressed in MGs/MΦs within ischemic areas after stroke.

Double staining immunohistochemistry for CD36 and DP1 was performed to investigate whether CD36^+^ MGs/MΦs expressed DP1 (Figure 5H–N). Although most CD36^+^ MGs/MΦs within ischemic areas co-expressed DP1 (Figure 5I,J,L–N), CD36^+^DP1^+^ cells were rarely observed in the contralateral side of nonischemic areas of the cortex (Figure 5K). These findings indicated that activated MGs/MΦs within ischemic areas co-expressed DP1 and CD36.

### 3.6. CD36^+^ MGs/MΦs Within Ischemic Areas Phagocytosed Damaged Myelin Fragments

**Figure 6:**
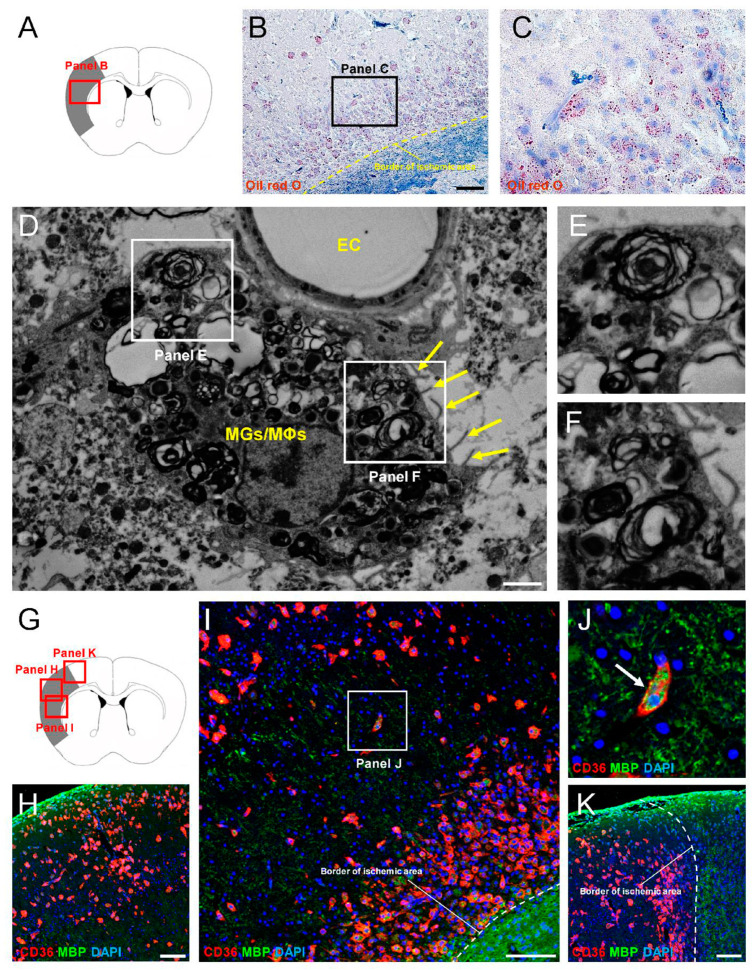
(**A**–**C**) Oil red O staining at 7 days post-stroke. Many oil red O^+^ cells were observed within ischemic areas. (**D**–**F**) Electron microscopy at 7 days post-stroke showed that MGs/MΦs with microvilli ((**D**), arrows) contained fragments of myelin-like structures (**E**,**F**). (**G**–**K**) Immunohistochemistry for CD36 and MBP. Although MBP^+^ cells keeping their original arrangement disappeared from the ischemic areas (**H**–**K**), MBP^+^ dots were observed in CD36^+^ MGs/MΦs in ischemic areas ((**J**), arrow). Scale bars: (**B**) 200 µm; (**D**) 2 µm; (**H**,**I**,**K**) 100 µm. Abbreviations: MBP, myelin basic protein; MGs/MΦs, microglia/macrophages.

We examined whether CD36^+^ MGs/MΦs acted as phagocytic scavengers of debris following ischemic stroke. It was reported that fragments of damaged myelin persisted within ischemic areas for a long time [34]. Because myelin is abundant in lipid components [35], we stained brain tissue sections with oil red O at 7 days post-stroke (Figure 6A–C). Many oil red O^+^ cells were observed in ischemic areas (Figure 6B,C), suggesting the presence of damaged myelin.

Electron microscopy examination of samples obtained from ischemic areas at 7 days post-stroke showed that MGs/MΦs with microvilli contained fragments of myelin-like structures (Figure 6D–F). We then performed immunohistochemistry for CD36 and MBP (Figure 6G–K), revealing that, although many MBP^+^ cells disappeared from the ischemic areas (Figure 6H,I,K), MBP^+^ staining, which did not keep the original arrangement, was observed in ischemic areas even after stroke (Figure 6I,J). Notably, MBP^+^ dots were observed in CD36^+^ MGs/MΦs (Figure 6J), suggesting that this cell population phagocytosed damaged myelin fragments in ischemic areas.

### 3.7. PGD2 Increases Phagocytic Activities of CD36^+^ MGs/MΦs

**Figure 7:**
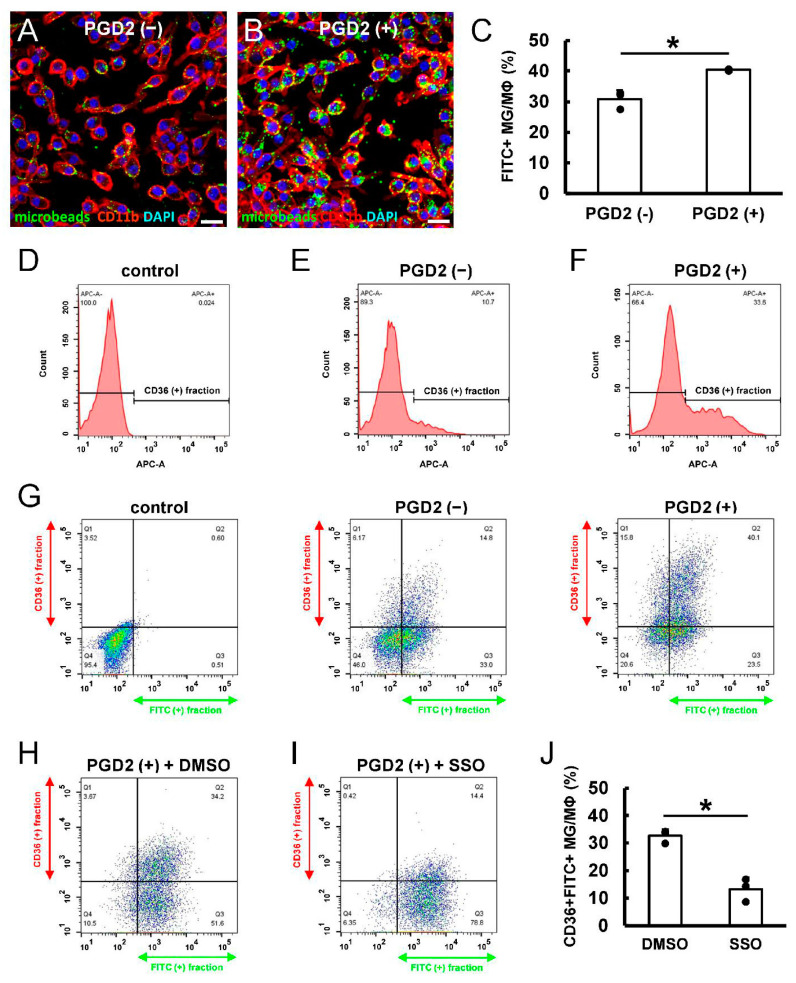
(**A**,**B**) Immunohistochemistry showing microbead uptake in CD11b^+^ MGs/MΦs treated without PGD2 (control) (**A**) or with PGD2 (**B**) (CD11b [(**A**,**B**): red]; DAPI [(**A**,**B**): blue]). (**C**) FACS analysis. The microbead uptake was significantly higher in PGD2-treated MGs/MΦs relative to the control MGs/MΦs. (**D**–**G**) FACS analysis shows the CD36^+^ fraction of MGs/MΦs lacking the primary antibody (control) (**D**), MGs/MΦs treated without PGD2 (**E**), and MGs/MΦs treated with PGD2 (**F**). FACS analysis shows the increased fraction of CD36^+^FITC^+^ MGs/MΦs after PGD2 treatment compared to no PGD2 treatment (**G**). (**H**–**J**) FACS analysis of MGs/MΦs after PGD2 treatment. Compared to DMSO-treated controls (**H**,**J**), the fraction of CD36^+^FITC^+^ MGs/MΦs was significantly decreased by additional treatment with CD36 inhibitor SSO (**I**,**J**). Scale bars: (**A**,**B**) 20 µm. * *p* < 0.05 between groups, *n* = 3 for each group (**C**,**J**). Abbreviations: DAPI, 4′,6-diamidino-2-phenylindole; DMSO, dimethyl sulfoxide; FACS, fluorescence-activated cell sorting; MGs/MΦs, microglia/macrophages; PGD2, prostaglandin D2; SSO, sulfo-N-succinimidyl oleate.

Our data showed that PGD2 promoted CD36^+^ MGs/MΦs production and that CD36^+^ MGs/MΦs potentially functioned as scavengers of myelin fragments in ischemic areas. Therefore, we next investigated whether PGD2 increased the phagocytic activities of MGs/MΦs by incubating them without or with PGD2 for 3 days. Then, they were treated with FITC-labeled microbeads for 2 h. Immunohistochemistry showed the uptake of FITC^+^ microbeads in both control CD11b^+^ MGs/MΦs and PGD2-treated CD11b^+^ MGs/MΦs, and FITC^+^ microbead uptake was obviously increased in PGD2-treated CD11b^+^ MGs/MΦs compared to the control (Figure 7A,B). FACS analysis demonstrated that the population of FITC^+^ cells was significantly increased in PGD2-treated MGs/MΦs compared to control MGs/MΦs (Figure 7C). These results confirmed that PGD2 increased phagocytic activities in MGs/MΦs.

Next, we investigated whether PGD2 treatment increased phagocytic activities in CD36^+^ MGs/MΦs. FACS analysis revealed that CD36^+^ populations were not observed in MGs/MΦs without CD36 primary antibody treatment (control) (Figure 7D). In contrast, CD36^+^ populations were observed in MGs/MΦs after treatment with CD36 primary antibody (Figure 7E,F), and CD36^+^ populations were increased in PGD2-treated MGs/MΦs (Figure 7F) compared to MGs/MΦs that did not receive PGD2 treatment (Figure 7E). Furthermore, FACS analysis for CD36 and FITC showed that the population of CD36^+^FITC^+^ MGs/MΦs increased after PGD2 treatment compared to cells that did not receive PGD2 treatment (Figure 7G). These findings indicated that PGD2 accelerated phagocytic activities of CD36^+^ MGs/MΦs. However, FACS analysis for CD36 and FITC showed that, compared to DMSO treatment (controls) (Figure 7H,J), the population of CD36^+^FITC^+^ MGs/MΦs after PGD2 treatment was significantly decreased following the addition of CD36 inhibitor SSO (Figure 7I,J).

## 4. Discussion

This is the first report showing that the L-PGDS–PGD2–DP1 axis has the potential to regulate the function of scavengers of waste products after ischemic stroke.

Cell death frequently occurs under normal physiological conditions during brain development [36]. Brain cell death also occurs due to various pathological conditions, such as neurodegenerative disease (e.g., AD), traumatic brain injuries, and ischemic stroke. Following cell death, the cells are rapidly removed by phagocytosis [37], and injured brains proceed to repair the neural cell damage. Although the reparative mechanism of injured brain tissue remains unclear, we previously demonstrated the activation of regionally derived stem cells at the sites of brain injuries and their involvement in neural regeneration [7,26]. However, severe brain injuries, such as ischemic stroke, produce excessive amounts of cellular debris and waste products (e.g., injured axons and myelin), which take a long time to clear. This prolonged phagocytic process results in physical and/or molecular barriers to neural regeneration through several mechanisms (e.g., preventing the outgrowth of newly generated neural cells and inducing long-lasting proinflammatory responses). Therefore, the ability to regulate phagocytosis would be important for not only brain development and maintenance under normal physiological conditions but also neural regeneration under pathological conditions.

L-PGDS is expressed in leptomeningeal cells in physiologically normal brains [6]. Although the L-PGDS expression patterns in brains with pathological conditions remain unclear, the present study demonstrated increased L-PGDS levels in the leptomeninges of ischemic areas and detected L-PGDS in pericytes in the leptomeninges. L-PGDS functions as a chaperone for waste products, such as amyloid-β [2,3] and biliverdin [4], in brains with pathological conditions. Thus, it is possible that brain pericytes expressing L-PGDS directly act as scavengers of debris produced after a brain injury.

L-PGDS produces PGD2 from PGH2 [10]. Although it is unclear which cell types produce PGD2 and PGH2 in the brain, it was reported that PGD2 increased following a brain injury, such as ischemic stroke [13,14,15], and that endothelial cells produced PGH2 [38]. Vascular lineage cells, such as pericytes and endothelial cells, are resistant to ischemia/hypoxia, enabling their survival within ischemic areas even after permanent ischemic stroke [7]. Thus, pericytes and endothelial cells may be sources of L-PGDS and PGH2, respectively, resulting in PGD2 production at sites of brain injuries.

PGD2 binds to PGD2 receptors DP1 and DP2 [16]. DP1 is expressed in MGs/MΦs [17,18,19], astrocytes [20], and neurons [21], while DP2 is expressed in astrocytes [17] but not in MGs/MΦs [22]. In the present study, we observed significant differences between the upregulation of gene expression levels of DP1 in ischemic areas compared to the contralateral side of nonischemic areas. In contrast, the gene expression levels of DP2 did not differ significantly between the ischemic areas and the contralateral side of nonischemic areas. Although we do not fully understand the reason, it has been established that neural lineage cells, such as neurons and astrocytes, are extremely vulnerable to ischemia/hypoxia and that ischemic areas become occupied with inflammatory cells, such as MGs/MΦs, after ischemic stroke [7]. Therefore, upregulated DP1 expression within ischemic areas may be attributed to the increased numbers of MGs/MΦs. This notion is supported by our study findings of high levels of DP1 expression in activated MGs/MΦs in brains with pathological conditions and is also consistent with previous reports [18,19].

Although the precise roles of receptors DP1 and DP2 in the brain remain unclear, DP1 stimulation by the DP1-selective agonist BW245C exerts a cytoprotective effect on neuronal cells. Conversely, DP2 activation promotes neuronal loss [39]. Additionally, the L-PGDS–PGD2–DP1 axis exerts beneficial effects on various pathological conditions in the brain, such as ischemic stroke [15,23,24,25], while the L-PGDS–PGD2–DP2 axis exerts negative effects on brain injuries [40]. It has also been established that the L-PGDS–PGD2–DP1 axis in the brain regulates sleep [41]. Therefore, the L-PGDS–PGD2–DP1 axis may be important for brain maintenance under not only pathological conditions but also normal physiological conditions.

The precise roles of the L-PGDS–PGD2–DP1 axis in brains with pathological conditions remain unclear. However, our current study demonstrated that PGD2 treatment of MGs/MΦs upregulated the expression of scavenger marker CD36, which was co-expressed with DP1. Additionally, PGD2-treated MGs/MΦs showed increased phagocytic activities in experiments assessing the uptake of FITC-labeled microbeads in vitro. Thus, further studies are warranted to clarify whether CD36^+^ MGs/MΦs serve as scavengers in ischemic areas in vivo. Our present study revealed that CD36^+^ MGs/MΦs within ischemic areas phagocytosed myelin fragments. Furthermore, there is increasing evidence that CD36^+^ MGs/MΦs promote the phagocytosis of myelin debris after various brain injuries, thereby reducing neuroinflammation [42] and promoting remyelination [43]. Moreover, it was reported that CD36 in MGs/MΦs contributed to phagocytosis during the resolution phase of ischemic stroke [33]. Thus, it is possible that CD36^+^ MGs/MΦs regulated by the L-PGDS–PGD2–DP1 axis serve as scavengers in brains with pathological conditions, such as ischemic stroke. Nevertheless, the precise role and function of CD36^+^ MGs/MΦs after brain injuries require elucidation in future studies, including human samples.

In conclusion, our study demonstrates for the first time that the L-PGDS–PGD2–DP1 axis regulates phagocytic activities of CD36^+^ MGs/MΦs that are exclusively present within ischemic areas. Thus, the administration of bioactive molecules that mediate the L-PGDS–PGD2–DP1 axis may be a potential therapeutic strategy for regulating the clearance of cellular debris following brain injuries, such as ischemic stroke.

## Data Availability

The data supporting this article will be shared by the corresponding author upon reasonable request.
